# Frequent occurrence of postbreakfast syncope due to carotid sinus syndrome after surgery for hypopharyngeal cancer: A case report

**DOI:** 10.1097/MD.0000000000025959

**Published:** 2021-05-21

**Authors:** Yuya Ando, Kenichi Hashimoto, Azusa Sano, Naoya Fujita, Rempei Yanagawa, Yosuke Ono, Yasuhiro Obuchi, Daisuke Tatsushima, Shun Watanabe, Masayuki Tomifuji, Yuji Tanaka

**Affiliations:** a Department of General Medicine, National Defense Medical College, Tokorozawa, Saitama; b Department of Family Medicine, Graduate School of Medical and Dental Sciences, Tokyo Medical and Dental University, Tokyo; c Department of Otolaryngology, National Defense Medical College, Tokorozawa, Saitama, Japan.

**Keywords:** carotid sinus syndrome, heart rate variability analysis, hypopharyngeal cancer, syncope, tilt table test

## Abstract

**Rationale:**

Syncope often occurs in patients with advanced head and neck cancers due to the stimulation of the autonomic nervous system by the tumor. Here, we describe a case of frequent syncopal episodes after laryngopharyngectomy for hypopharyngeal cancer. As all syncopal episodes were observed during the forenoon, we also evaluated the heart rate variability using ambulatory electrocardiography to determine why the syncopal episodes occurred during a specified period of the day.

**Patient concerns:**

A 73-year-old Japanese man who underwent laryngopharyngectomy for recurrent hypopharyngeal cancer started experiencing frequent episodes of loss of consciousness that occurred during the same time period (10:00–12:00). He had never experienced syncopal episodes before the operation. From 23 to 41 days postoperatively, he experienced 9 syncopal episodes that occurred regardless of his posture.

**Diagnoses:**

Pharyngo-esophagoscopy revealed an anastomotic stricture between the free jejunum graft and the upper esophagus. Swallowing videofluoroscopy confirmed the dilatation of the jejunal autograft and a foreign body stuck on the oral side of the anastomosis. Contrast-enhanced computed tomography revealed that the carotid artery was slightly compressed by the edematous free jejunum. The patient was diagnosed with carotid sinus syndrome (CSS) as the free jejunum was dilated when consuming breakfast, which may have caused carotid sinus hypersensitivity and induced a medullary reflex.

**Interventions:**

Administration of disopyramide was effective in preventing syncope. Heart rate variability analysis using ambulatory electrocardiography showed that parasympathetic dominancy shifted to sympathetic dominancy during 10:00 to 12:00. The significant time regularity of the syncopal episodes may have been affected by modified diurnal variation in autonomic tone activity.

**Outcomes:**

After the surgical release and re-anastomosis of the pharyngoesophageal stenosis via an open-neck approach, no recurrent episodes of syncope were reported.

**Lessons:**

We reported a case of frequent syncopal episodes limited to the forenoon due to CSS after surgery for hypopharyngeal carcinoma. The patient was treated with anticholinergics followed by the release and re-anastomosis of the pharyngoesophageal stenosis. When syncope occurs after surgery for head and neck lesions, CSS due to postoperative structural changes should be considered as a differential diagnosis of syncope.

## 1 Introduction

Syncope is one of the comorbidities associated with untreated head and neck cancers.^[1–8]^ Tumor progression or invasion can lead to carotid sinus syndrome (CSS). Several researchers have mentioned that the medullary reflex is invoked by mechanical compression of the carotid sinus or glossopharyngeal nerve by the tumor.^[2–5]^ These reports suggest that such episodes of syncope occur only before treatment, with their incidence decreasing after interventions, such as surgery, radiation therapy, and chemotherapy. Here, we report a case of syncope caused by CSS despite treatment with hypopharyngeal surgery. The syncopal episodes were observed only during the period from 10:00 to 12:00.

## 2 Case presentation

### 2.1 Patient Information

A-73-year-old retired male presented with local recurrence of hypopharyngeal squamous cell cancer. He had undergone radiotherapy (70 Gy) 2 years before presentation and had no coronary risk factors, such as smoking history. His past medical history included a hypopharyngeal neoplasm originating from the postcricoid region or left pyriform sinus clinically staged as rT1N0M0. He was admitted to our facility for radical surgery.

### 2.2 Clinical findings

His body weight and height were 59 kg and 169 cm, respectively, corresponding to a body mass index of 20.7 kg/m^2^. On admission, he was afebrile, his oxygen saturation with room air, heart rate, and blood pressure were 98%, 68 beats/min, and 108/68 mm Hg, respectively. The 12-lead electrocardiogram showed normal findings.

The patient underwent laryngoesophagopharyngectomy (first surgery), left cervical lymph node dissection, thyroidectomy, and esophageal reconstruction with free jejunal autograft. Early postoperative complications were not observed. Though the patient had never experienced syncopal episodes, he had experienced a total of 9 episodes of vasodepressor-type syncope from 23 to 41 days postoperation. Surprisingly, all syncopal episodes occurred during 10:00 to 12:00 (Table 1).

**Table 1:** Characteristics of all the syncopal episodes.

No.	Days after the 1st surgery	Onset time	Minimum BP (mm Hg)	Minimum HR (/min)	Syncope or presyncope	Type of CSS	Posture	Medication
1	23	10:02	58/35	40	Presyncope	Vasodepressor	Decubitus	
2	25	10:19	62/40	40	Syncope	Vasodepressor	Decubitus	
3	26	10:53	70/–	38	Syncope	Vasodepressor	Sitting	Midodrine hydrochloride 4 mg/d
4	28	12:37	98/39	30	Syncope	Vasodepressor	Sitting	Fludrocortisone acetate 0.1 mg/d
5	29	10:53	50/40	38	Presyncope	Vasodepressor	Decubitus	
6	32	12:26	55/–	27	Syncope	Vasodepressor	Unknown	
7	33	10:58	N/A	31	Presyncope	Vasodepressor	Sitting	
8	37	10:45	40/–	19	Presyncope	Vasodepressor	Sitting	Disopyramide 200 mg/d
9	41	10:37	62/35	32	Presyncope	Vasodepressor	Decubitus	

No convulsions were seen during all 9 episodes, which occurred in the hospital room.

### 2.3 Diagnostic assessment

**Figure 1:**
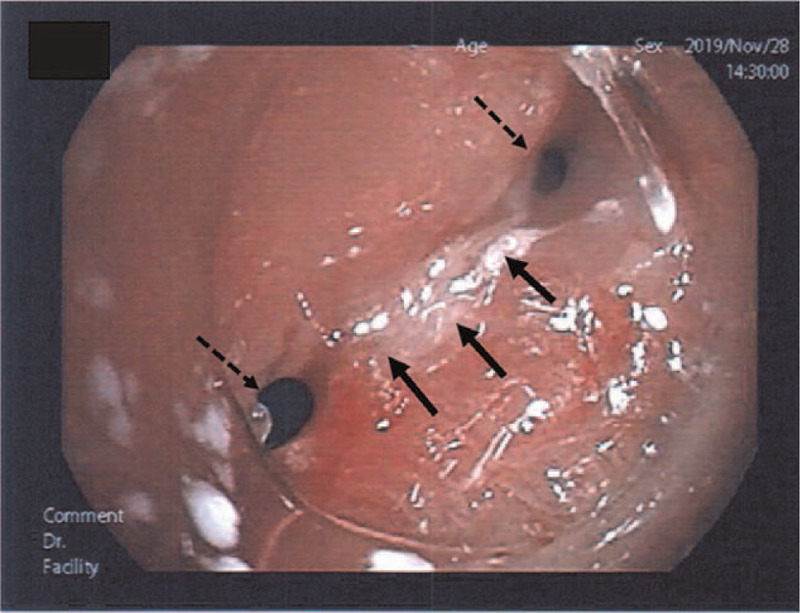
Pharyngo-esophagoscopy findings at the upper esophagus-free jejunum anastomosis after pharyngolaryngoesophagectomy. An anastomotic stenosis is seen between the free jejunum and upper esophagus (arrows). The narrow, divided space of the anastomosis makes it difficult for the food to pass through (dotted arrows).

**Figure 2:**
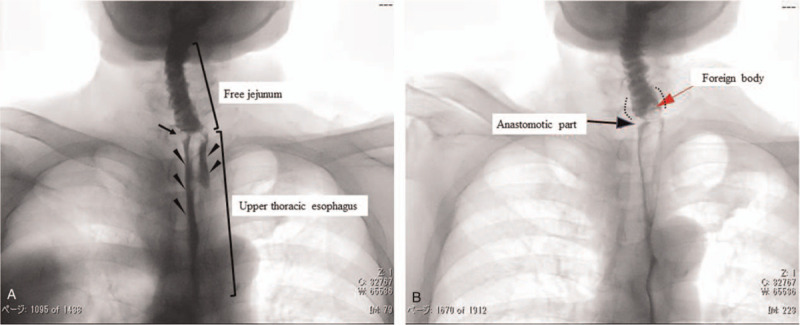
Swallowing videofluoroscopy (VF) findings. Swallowing videofluoroscopy (VF) shows that the contrast agent stagnated in the mouth, the inflow into the stomach was delayed, and the contrast agent was divided into 2 central areas due to the anastomosis (A) (Triangle 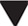). Swallowing VF examination performed when the patient felt a discomfort in the larynx. The foreign body (red arrow) stagnating in the anastomosis (black arrows) and producing a transparent image. The free jejunum on the oral side is dilated (dotted line) (B).

**Figure 3:**
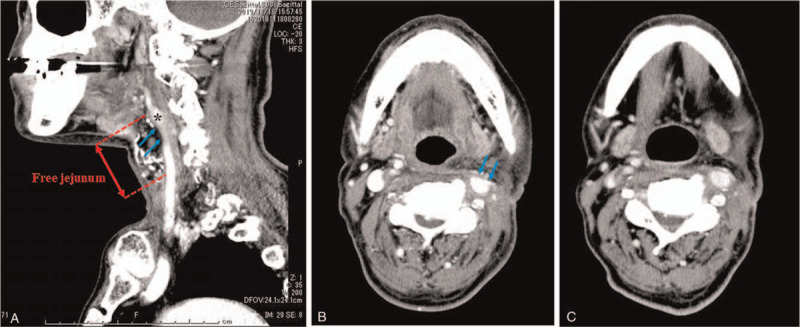
Head and neck CT at the carotid sinus level (27 d after the first surgery). After pharyngolaryngoesophagectomy with free jejunum replacement, the carotid artery is slightly compressed by the edematous free jejunum graft (Blue arrows) (A, sagittal view; B, axial CT). After the surgical release and re-anastomosis of the pharyngoesophageal stenosis via an open-neck approach, the free jejunum edema improved. The contact between the left carotid sinus and the free jejunum is reduced (78 d after the first surgery) (C). ∗Indicates the carotid sinus. CT = computed tomography.

Carotid sinus massage (CSM) testing was negative. Tilt table test (TTT) was positive for vasodepressor-type presyncope accompanied by nausea, which is clinically identical to syncope. Myocardial scintigraphy showed no evidence of coronary artery stenosis, whereas pharyngo-esophagoscopy revealed an anastomotic stenosis between the free jejunal autograft and upper esophagus (Fig. 1). Swallowing videofluoroscopy (VF) examination showed a delay in contrast medium movement into the stomach due to the anastomotic stenosis, resulting in the double flow stream image of the contrast medium distal to the stenosis (Fig. 2A). VF was performed again when the patient complained of throat discomfort. Dilatation of the jejunal graft was observed due to a foreign body that was stuck in the oral side of the anastomosis (Fig. 2B). Contrast-enhanced computed tomography (CT) indicated that the jejunal autograft slightly compressed the carotid artery (Fig. 3A and B). The syncopal episodes were attributed to CSS caused by dilatation of the edematous autograft, which induced obstruction at the stenotic site due to food debris. However, a possible contribution of vasovagal syncope (VVS) to the occurrence of syncope could not be ruled out at that time.

### 2.4 Therapeutic intervention

Oral intake of midodrine hydrochloride 4 mg/d and fludrocortisone acetate 0.1 mg/d was not effective in preventing syncope. Oral intake of disopyramide 200 mg/d was started from 33 days after the first surgery, after which the incidence of syncope markedly decreased. Although the patient had a final syncopal episode on the 41st day after the first surgery, the disopyramide tablets did not pass through the anastomotic stricture, which led to them not being absorbed in the digestive tract on that day. Fifty days after the first surgery, surgical release and re-anastomosis of the pharyngoesophageal stenosis (the second surgery) were performed via an open-neck approach. After this procedure, no recurrence of syncopal episodes has been reported even without disopyramide administration.

**Figure 4:**
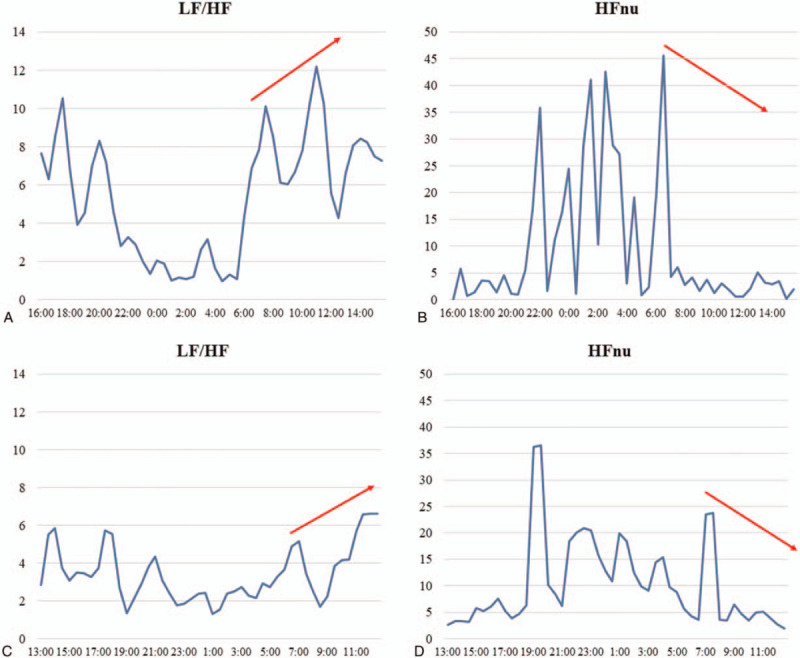
Diurnal HRV variation. Before endoscopic adhesiolysis, the LF/HF ratio gradually increased from 6:00 and peaked at 10:00 to 11:00 (A) (45 d after admission). Conversely, HFnu steadily declined at the same time (B). The diurnal variation of the autonomic nervous activity remains similar even after endoscopic adhesiolysis (C, D) (94 d after the first operation). HFnu = international normalized unit of high frequency area, HRV = heart rate variability, LF/HF = low frequency/high frequency ratio.

Heart rate variability (HRV) analysis was performed through ambulatory electrocardiography recording (SCM 8000, Fukuda Denshi, Tokyo, Japan) for the evaluation of autonomic nervous activity to investigate the cause of the time regularity of the syncopal episodes. The HRV analysis parameters were obtained from the frequency analysis of the RR interval of the cardiac cycle using the fast Fourier transform method. The low (LF) and high (HF) frequency ranges were defined as 0.04 to 0.15 and 0.15 to 0.40 Hz, respectively.^[9]^ The LF/HF ratio was also calculated. The international normalized unit of high frequency area (HFnu) was calculated as HF/(HF + LF).^[9]^ The HRV power value was automatically measured every 5 minutes, and the average values of all parameters were calculated every 30 minutes (Fig. 4A–D). In general, LH/HF reflects both sympathetic and parasympathetic nerve activities, whereas HFnu mainly indicates a parasympathetic nervous tone. Before the second surgery, LF/HF gradually increased from 6:00 and peaked at 10:00 to 1:00 (Fig. 4A). At the same time, HFnu declined steadily (Fig. 4B). The diurnal variation trends in autonomic nervous activity evaluated by HRV remained similar even after the second surgery (Fig. 4C and D). The patient was discharged at 65 days after the first surgery in a good postoperative state.

### 2.5 Follow-up and outcomes

A head and neck contrast CT performed at 14 days after discharge showed an improvement in the free jejunal edema and compression of carotid artery (Fig. 3C). During the 4-month outpatient follow-up, the patient did not experience any syncope recurrences.

## 3 Discussion

Although the patient did not have a history of syncopal episodes, he had syncope/presyncope due to CSS, which occurred after surgery for hypopharyngeal cancer. In addition, syncopal/presyncopal seizures had a very distinctive feature, in that they occurred only during 10:00 to 12:00. Oral midodrine hydrochloride and fludrocortisone were not effective in preventing syncope; however, disopyramide administration ended the syncopal episodes. After reoperation, syncope/presyncope disappeared. HRV analysis using 24-hour ambulatory electrocardiography helped us unveil the mechanism by which the episodes occurred within a limited time period.

Wang et al,^[5]^ reported that all 9 cases of syncope due to CSS accompanied by pharyngeal cancer in their study occurred only before treatment. To the best of our knowledge, there are few reports of syncope occurring postoperatively. Previously, 2 mechanisms of CSS caused by a neck tumor have been put forward. The first mechanism is direct mechanical compression of the carotid sinus or compression of the glossopharyngeal nerve due to the presence of a tumor in the parapharyngeal space.^[3]^ Second, the stimulation by the tumor may lead to depolarization and increased nerve firing, triggering the carotid sinus reflex.^[10]^ In any case, the afferent stimulus by the glossopharyngeal nerve induces a reflex in the medulla oblongata nuclei, which is then transmitted to the heart branch of the parasympathetic nerve, leading to syncope. In this case, the precise pathophysiological mechanism of syncope was unknown. However, we speculate the following mechanism. First, the anastomosis was narrowed in the lower portion of the carotid sinus due to a complicated surgical procedure for hypopharyngeal cancer (Fig. 2). The baroreceptors in the carotid sinus might be slightly compressed by the edematous jejunum graft dilated by food debris. Second, inflammation or adhesion of the surgical field could lead to depolarization and increased nerve firing in the sinus reflex pathway, inducing a reflex in the medulla oblongata nuclei.

VVS could not be ruled out as the pathophysiological mechanism for some of the 9 syncopal episodes. There was no direct compression of the carotid sinus since some distances remained between the anastomotic portion and the carotid sinus (Fig. 3A). Otherwise, typically, a patient with CSS shows positive CSM and negative TTT findings.^[11]^ These typical results were not consistent with the results in this case. However, it has been reported that the false negative rate of CSM was 34% and the false positive rate of TTT was 18% in patients with CSS.^[12,13]^ Ultimately, syncopal episodes disappeared after the second surgery even without the use of disopyramide. Given the fact that the syncope occurred regardless of the patient's posture and the CT and VF findings, CSS and VVS could be the main and secondary causes of syncope, respectively.

Oral administration of disopyramide successfully improved the patient's symptoms in this case. A possible mechanism to explain the effectiveness of disopyramide in this case is that the increased parasympathetic activity in the centrifugal tract was suppressed by its anticholinergic effect.^[14]^ However, disopyramide is not a clinically established treatment for autonomic dysregulation. Disopyramide has received a class III recommendation for preventing syncope in patients with CSS in previous guidelines.^[14]^ Furthermore, disopyramide has been reported to be effective only in a small number of cases in patients (n = 11) with tilt-induced hypotension-bradycardia.^[15]^

CSS is classified into the following 3 types according to the response to CSM: cardioinhibitory, vasodepressor, and mixed CSS.^[16,17]^ Cardioinhibitory CSS is characterized by cardiac arrest for >3 seconds with CSM and a drop in systolic blood pressure of <50 mm Hg, whereas vasodepressor CSS does not respond markedly to CSM but causes a drop in systolic blood pressure of >50 mm Hg. Mixed CSS is characterized by symptoms of both previously described types. All syncopal episodes in this case were attributed to vasodepressor CSS. Various studies have mentioned that pacemaker implantation is effective for patients with cardioinhibitory and mixed CSS,^[18]^ whereas no effective treatment has been established for vasodepressor CSS. Therefore, our findings show that disopyramide may be useful for preventing syncope in patients with vasodepressor CSS.

The patient had repeated syncope/presyncope limited to the same time period between 10:00 and 12:00. More precisely, 7 out of the 9 syncopal episodes occurred around 10:00; the remaining 2 occurred around 12:00 (Table 1). We propose the following mechanism for explaining why the syncopal episodes occurred within a limited period: the breakfast ingested in the morning accumulated for several hours at the free jejunum, slightly compressing the carotid artery or leading to depolarization of the nerves in the sinus reflex pathway during 10:00 to 12:00. Data from the HRV analysis suggest that the autonomic nerve activity shift from parasympathetic to sympathetic predominance tends to occur at 10:00 to 12:00 (Fig. 4A and B). Subsequently, sympathetic over activity causes syncope via the medullary reflex due to the diurnal variation in HRV. In this case, the diurnal variation in HRV was similar to the previously reported HRV in healthy subjects.^[19,20]^ Miranda et al reported that elevation in the LF/HF ratio and a decrease in HF were observed when the head was up during the TTT in patients with syncope.^[21]^ Moreover, they reported that this phenomenon was more pronounced in these patients than in healthy subjects. The autonomic nerve balance in this case changed during the 10:00 to 12:00 period, which is a similar situation to what occurs during tilt-up in the TTT and might lead to syncope. Nevertheless, the precise mechanism behind why the syncopal episodes occurred within a fixed time of the day is ultimately unknown. However, it seems clear that a type of autonomic imbalance was involved in the pathogenesis. Therefore, there is a possibility that diurnal variations in the autonomic nervous system may explain why all syncopal episodes occurred within a fixed time period.

## 4 Conclusion

We reported a case of frequent syncopal episodes limited to the postbreakfast period mainly due to postoperative CSS occurring after surgery for hypopharyngeal carcinoma; this was treated with anticholinergics followed by the release and re-anastomosis of the pharyngoesophageal stenosis. In patients with malignancies of the head and neck who experience frequent syncopal episodes, it is important to suspect CSS due to structural changes caused by reconstructive surgery even after treatment. HRV analysis suggested that all the syncopal episodes occurred within a limited time period partially due to the diurnal variation of autonomic nervous activity in the forenoon.

## Acknowledgments

The authors would like to thank Dr Yuji Kasamaki of Kanazawa Medical University Himi Municipal Hospital for interpreting the HRV findings and Dr Nishizaki of Kanto Gakuin University for interpreting the mechanism of syncope.

## Author contributions

**Conceptualization:** Yuya Ando, Kenichi Hashimoto, Azusa Sano, Naoya Fujita, Yosuke Ono, Yasuhiro Obuchi, Daisuke Tatsushima, Shun Watanabe, Masayuki Tomifuji, Yuji Tanaka.

**Data curation:** Yuya Ando, Kenichi Hashimoto, Yasuhiro Obuchi, Masayuki Tomifuji.

**Formal analysis:** Yuya Ando, Kenichi Hashimoto.

**Investigation:** Yuya Ando, Kenichi Hashimoto, Azusa Sano, Naoya Fujita, Rempei Yanagawa, Yosuke Ono, Daisuke Tatsushima, Shun Watanabe, Masayuki Tomifuji.

**Methodology:** Kenichi Hashimoto.

**Project administration:** Kenichi Hashimoto, Yasuhiro Obuchi.

**Supervision:** Kenichi Hashimoto, Naoya Fujita, Daisuke Tatsushima, Masayuki Tomifuji, Yuji Tanaka.

**Visualization:** Kenichi Hashimoto.

**Writing – original draft:** Yuya Ando, Kenichi Hashimoto.

**Writing – review & editing:** Yuya Ando, Kenichi Hashimoto, Azusa Sano, Naoya Fujita, Rempei Yanagawa, Yosuke Ono, Daisuke Tatsushima, Shun Watanabe, Masayuki Tomifuji, Yuji Tanaka.
